# Sulforaphane attenuates EGFR signaling in NSCLC cells

**DOI:** 10.1186/s12929-015-0139-x

**Published:** 2015-06-03

**Authors:** Chi-Yuan Chen, Zhu-Yun Yu, Yen-Shu Chuang, Rui-Mei Huang, Tzu-Chien V Wang

**Affiliations:** Research Center for Industry of Human Ecology, Chang Gung University of Science and Technology, Kwei-San, Tao-Yuan 333 Taiwan; Graduate Institute of Health Industry Technology, Chang Gung University of Science and Technology, Kwei-San, Tao-Yuan 333 Taiwan; Department of Molecular and Cellular Biology, College of Medicine, Chang Gung University, Kwei-San, Tao-Yuan 333 Taiwan

**Keywords:** Sulforaphane, EGFR, Lung cancer, HSP90, TKI

## Abstract

**Background:**

EGFR, a receptor tyrosine kinase (RTK), is frequently overexpressed and mutated in non-small cell lung cancer (NSCLC). Tyrosine kinase inhibitors (TKIs) have been widely used in the treatment of many cancers, including NSCLC. However, intrinsic and acquired resistance to TKI remains a common obstacle. One strategy that may help overcome EGFR-TKI resistance is to target EGFR for degradation. As EGFR is a client protein of heat-shock protein 90 (HSP90) and sulforaphane is known to functionally regulate HSP90, we hypothesized that sulforaphane could attenuate EGFR-related signaling and potentially be used to treat NSCLC.

**Results:**

Our study revealed that sulforaphane displayed antitumor activity against NSCLC cells both *in vitro* and *in vivo*. The sensitivity of NSCLC cells to sulforaphane appeared to positively correlate with the inhibition of EGFR-related signaling, which was attributed to the increased proteasomal degradation of EGFR. Combined treatment of NSCLC cells with sulforaphane plus another HSP90 inhibitor (17-AAG) enhanced the inhibition of EGFR-related signaling both *in vitro* and *in vivo*.

**Conclusions:**

We have shown that sulforaphane is a novel inhibitory modulator of EGFR expression and is effective in inhibiting the tumor growth of EGFR*-*TKI-resistant NSCLC cells. Our findings suggest that sulforaphane should be further explored for its potential clinical applications against NSCLC.

## Background

Lung cancer is the most common cause of cancer deaths worldwide, and non-small cell lung cancer (NSCLC) is the major dominant cell type [1]. The epidermal growth factor receptor (EGFR) is highly expressed in many human tumors, including NSCLC. The intracellular signaling pathways activated by EGFR, which include the PI3K/AKT/mTOR, RAS/RAF/MAPK and JAK/STAT pathways, play central roles in controlling cell survival, growth and proliferation [2]. Inhibition of EGFR signaling, therefore, has been widely explored for its therapeutic potential against many different cancers [3]. Treatment with EGFR-tyrosine kinase inhibitor (TKI) has led to dramatic clinical improvement in some patients with NSCLC [4], but intrinsic and acquired resistance to EGFR-TKI is common [5,6]. One potential strategy for addressing EGFR-TKI resistance is to target EGFR for degradation. As receptor tyrosine kinases (RTKs) comprise the largest category of heat shock protein 90 (HSP90) client proteins [7], one strategy aimed at targeting RTKs for degradation is to inhibit HSP90. Many inhibitors of HSP90 have been developed and some of them, such as 17-N-allylamino-17-demethoxygeldanamycin (17-AAG), are currently undergoing clinical trials as potential anticancer drugs [8]. However, 17-AAG is poorly soluble and suffers from low oral bioavailability, metabolism issues and hepatotoxicity [8].

Natural products have been the major source of the currently available anticancer drugs, and identifying their active chemical ingredients and deducing the molecular targets of such ingredients is viewed as an attractive approach for drug development. Sulforaphane, which was first identified in broccoli sprouts and is present at high concentrations in most cruciferous vegetables [9], has been shown to be potentially effective at moderating multiple cellular targets involved in cancer development, leading to the repression of cancer cell proliferation, stimulation of cancer cell apoptosis, and inhibition of tumor progression and metastasis [10,11]. Combined treatment with doxorubicin and sulforaphane has been shown to reverse the doxorubicin-resistant phenotype in mutant mouse fibroblasts [12], while combined treatment with epigallocatechin gallate (EGCG) and sulforaphane has been shown to induce apoptosis in paclitaxel-resistant ovarian cancer cell lines [13]. Therefore, sulforaphane appears to effectively counteract the drug-resistant phenotypes of several cancers. In view of the recent finding that sulforaphane can inhibit HSP90 function in prostate and pancreatic cancer cell lines [14-16], we hypothesized that sulforaphane may be a novel EGFR-targeting therapeutic agent that could be used for the treatment of NSCLC. In this study, we examined the EGFR modulation activity and antitumor potential of sulforaphane in several NSCLC cell lines which harboring wild-type or mutant EGFR. Our results indicate that sulforaphane is a novel modulator of EGFR and is effective in inhibiting the tumor growth of EGFR*-*TKI-resistant NSCLC cells.

## Methods

### Cell lines and culture

The NSCLC-derived cell lines, A549, H1975 and H3255, were obtained from the American Type Culture Collection (Manassas, VA, USA). PC9/gef was a gefitinib-resistant cell line that had been selected from parental PC9 cells by continuous exposure to an increasing dosage of gefitinib over ~ six months, as previously described [17]. The human NSCLC cell line, CL1-5, was as described previously [18]. A549 and CL1-5 cells express wild-type EGFR; PC9 cells contain a deletion in exon 19 of *EGFR*; and H1975 and H3255 cells harbor two mutations (L858R and T790M) and a single mutation (L858R), respectively, in *EGFR*. The primary normal human fibroblasts (HFB) were kindly provided by Dr. Pan-Chyr Yang (National Taiwan University, Taipei, Taiwan). All cells were cultivated in RPMI-1640 medium containing 10% fetal bovine serum (FBS), 2 mM sodium pyruvate, 100 U/ml penicillin and 100 U/ml streptomycin. Cells were grown at 37°C in a humidified incubator containing 5% CO_2_.

### Antibodies, oligonucleotides, and reagents

Culture media, chemical compounds and FBS were purchased from Life Technologies (Grand Island, NY, USA). Antibodies against phospho-EGFR (Tyr1068), phospho-STAT3 (Tyr705) and phospho-AKT (Ser473) were purchased from Cell Signaling (Temecula, CA, USA). Antibodies against EGFR, STAT, AKT and β-actin were purchased from Santa Cruz Biotechnology (Santa Cruz, CA, USA). Sulforaphane, MG132 and cycloheximide were purchased from Sigma (St. Louis, MO, USA). 17-AAG was purchased from Calbiochem (Gibbstown, NJ, USA).

### Cell viability, synergy and Western blot analyses

Cell viability (MTT) assays, synergy and Western blotting were performed as described previously [19,20].

### Foci formation assay

Cells were plated to 6-well plates (100 cells per well) for 24 h and then treated with sulforaphane at the indicated concentrations for 6 days in complete culture medium. The treated cells were incubated in complete medium without sulforaphane for an additional 8 days, and then subjected to staining with 0.001% crystal violet. A foci was defined as a group of stained cells > 1 mm in diameter.

### Stability of EGFR analysis

Cells were treated with or without 10 μM sulforaphane for 12 h. Cells were then treated with cycloheximide (40 μg/ml) for 0, 2, 4, 6, and 8 h and the level of total EGFR (tEGFR) was determined by Western blotting.

### Subcutaneous xenograft-based animal studies

The *in vivo* antitumor activity of sulforaphane and/or 17-AAG against human NSCLC was studied using 6-week-old nude BALB/c nu/nu male mice (*n* = 6 per group). Animals were inoculated subcutaneously in the right flank with tumor cells (2 × 10^6^) in a volume of 100 μL on day 0. Mice were randomly divided into four groups on day 5 and treated with PBS as control, 10 μmol/kg sulforaphane, 25 mg/kg 17-AAG, or both sulforaphane and 17-AAG [15]. Sulforaphane was dissolved in PBS whereas 17-AAG was dissolved in 10% DMSO, 70% cremophor/ethanol (3:1), and 20% PBS [15]. Sulforphane was injected intratumorally five times per week. 17-AAG was administered intraperitoneally three times per week. Tumors were generally palpable at 5 days after inoculation. Drug treatment began at Day 5 and tumor volumes were measured (using a caliper and calculated as length x width^2^ × 0.5) twice weekly until 21 days after injection [21]. All animal experiments were performed in accordance with the guidelines for the Animal Care Ethics Commission of Chang Gung University under an approved animal protocol.

### Statistical analysis

The presented results are representative of three independent experiments with similar results. Statistical differences were evaluated using the Student’s *t*-test, and were considered significant at *p* < 0.05.

## Results

### Effects of sulforaphane on the viability and growth of human NSCLC cells *in vitro*

**Fig. 1:**
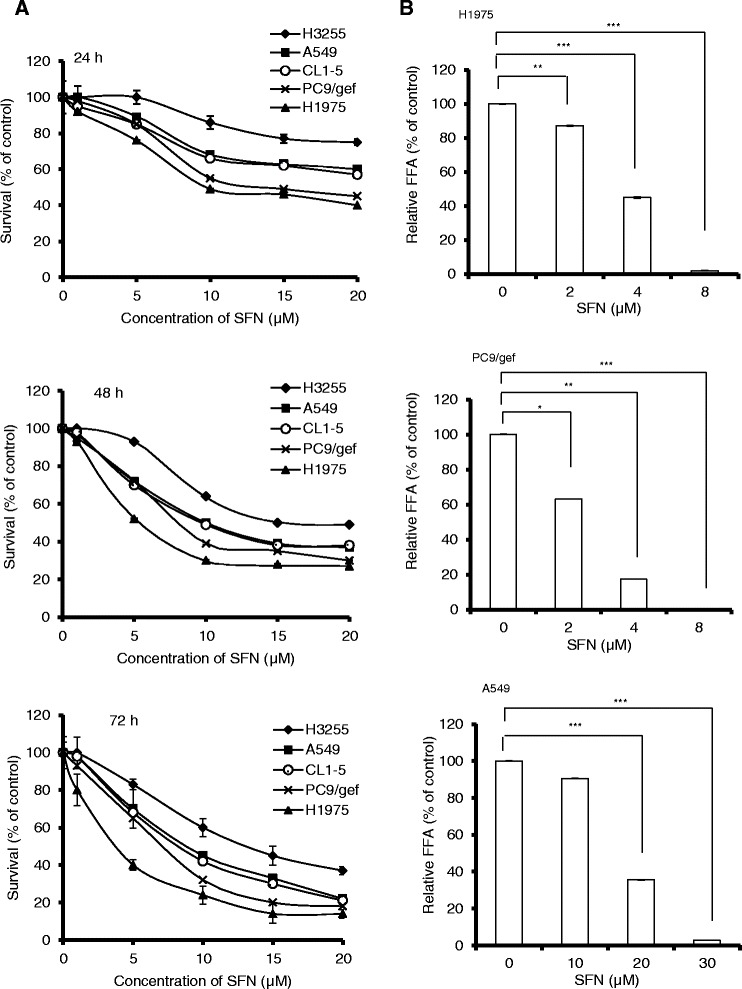
Effects of sulforaphane on the cell viability and clonogenic ability of NSCLC cells. (**a**) Time- and concentration-dependent inhibition of NSCLC cell viability by sulforaphane (SFN). Cells were treated with various concentrations of sulforaphane for 24 h (upper panel), 48 h (middle panel) and 72 h (lower panel), and cell viability was evaluated by MTT assay. (**b**) Inhibition of the clonogenic ability of NSCLC cells by sulforaphane. H1975 (upper panel), PC9/gef (middle panel) and A549 (lower panel) cells were treated with various concentrations of sulforaphane for 6 days and cultured for an additional 8 days in the absence of sulforaphane. The numbers of foci were scored, and the data are presented as relative foci-forming ability (FFA). Data are expressed as mean ± SD of three independent experiments. *p < 0*.*05; **p < 0*.*01; and ***p < 0*.*001, as analyzed with the unpaired *t*-test

To evaluate the antitumor effects of sulforaphane on NSCLC cells, the TKI-resistant (PC9/gef, H1975, A549, and CL1-5) and the TKI-sensitive (H3255) cells were treated with sulforaphane at 5–20 μM and their viability were measured by MTT assay. As shown in Fig. 1a, sulforaphane treatment remarkably reduced the viability of human NSCLC cells in a time- and concentration-dependent manner. Among the TKI-sensitive (H3255) and TKI-resistant (PC9/gef, H1975, A549, and CL1-5) NSCLC cells, H1975 cells exhibited the highest sensitivity to sulforaphane (Table 1 and Fig.1a). A primary normal human fibroblasts (HFB), on the other hand, were rather resistant to sulforaphane (Table 1). Next, we examined the effect of sulforaphane on the foci-forming ability of TKI-resistant (PC9/gef, H1975, and A549) NSCLC cells. As shown in Fig. 1b, the foci-forming ability of H1975 and PC9/gef cells was fully suppressed by sulforaphane at a concentration of 8 μM (Fig. 1b, upper and middle panels). In contrast, a much higher concentrations of sulforaphane (e.g., 30 μM) were required to produce the same inhibition of foci-forming ability in A549 cells (Fig. 1b, lower panel).

**Table 1:** The IC50 of sulforaphane for human NSCLC cells harboring different EGFR mutations

Cell line	EGFR mutation status	Sulforaphane
		IC50 (μM) ^a^
A549	Wild-type	10.2 ± 0.15
CL1-5	Wild-type	9.7 ± 0.33
H3255	L858R ^b^	14.5 ± 0.21
PC9/gef ^c^	Exon 19 del.	7.3 ± 0.25
H1975	L858R/T790M ^d^	5.9 ± 0.18
HFB	Wild-type	65.4 ± 0.29

^a^Cells were treated with various concentrations of sulforaphane for 48 h, and cell viability was determined by MTT assay. The IC50 values were calculated by non-linear regression analysis. Values are given as means ± standard deviation

^b^L858R substitution in exon 21

^c^PC9/gef is a gefitinib-resistant cell line that was selected from parental PC9 cells (which contain a deletion in exon 19 of *EGFR*) by continuous exposure to increasing doses of gefitinib over ~ 6 months

^d^L858R substitution in exon 21 and a secondary T790M substitution in exon 20 of *EGFR*

### Sulforaphane attenuates EGFR expression and EGFR signaling in NSCLC cell lines

**Fig. 2:**
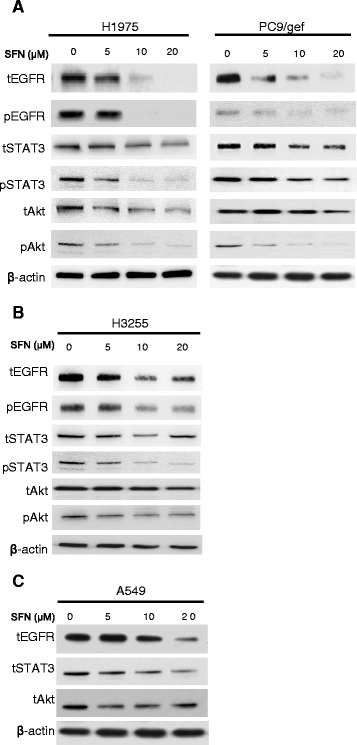
Effects of sulforaphane on EGFR-related signaling in NSCLC cells. H1975, PC9/gef (**a**), H3255 (**b**) and A549 (**c**) cells were treated with various concentrations of sulforaphane (SFN) for 24 h, and the levels of total (t) and phosphoylated (p) proteins in the cell lysates were determined by Western blotting. β-actin was used as the loading control. The data shown are representative of three experiments with similar results

Mutant EGFRs induce oncogenic effects by triggering downstream signaling and anti-apoptotic pathways, most markedly those modulated by signal transducer and activator of transcription (STAT) proteins and Akt [22]. Because sulforaphane appeared to exhibit a greater anti-proliferative effect in TKI-resistant NSCLC cells harboring mutant EGFR (H1975 and PC9/gef) than wild-type EGFR (A549) (Fig. 1 and Table 1), we hypothesized that suforaphane might interfere with the EGFR signaling. To test this postulate, we treated H1975 and PC9/gef cells with sulforaphane (0 – 20 μM) and examined EGFR-related signaling. As shown in Fig. 2, the activation of phospho-EGFR (pEGFR) and phospho-Akt (pAkt) were readily detected in untreated H1975 and PC9/gef cells, and were greatly reduced after treatment with sulforaphane. Furthermore, the phospho-STAT3 (pSTAT3) was also greatly reduced in sulforaphane-treated H1975 cells but not in sulforaphane-treated PC9/gef cells. The levels of p-EGFR, p-STAT3, and p-Akt were slightly reduced in sulforaphane-treated H3255 cells (Fig. 2b). In A549 cells, we were not able to detect pEGFR, pAkt or pSTAT3 in the untreated cells, and thus could not address the effect of sulforaphane on EGFR-related signaling in the absence of any stimulation. Nonetheless, the levels of total EGFR (tEGFR) were also reduced upon treatment with sulforaphane in A549 cells (Fig. 2c). Notably, however, tEGFR was more dramatically reduced in H1975 and PC9/gef cells compared to H3255 and A549 cells following sulforaphane treatment (Fig. 2).

### Sulforaphane potentially modulates EGFR expression by accelerating protein degradation in NSCLC cells

**Fig. 3:**
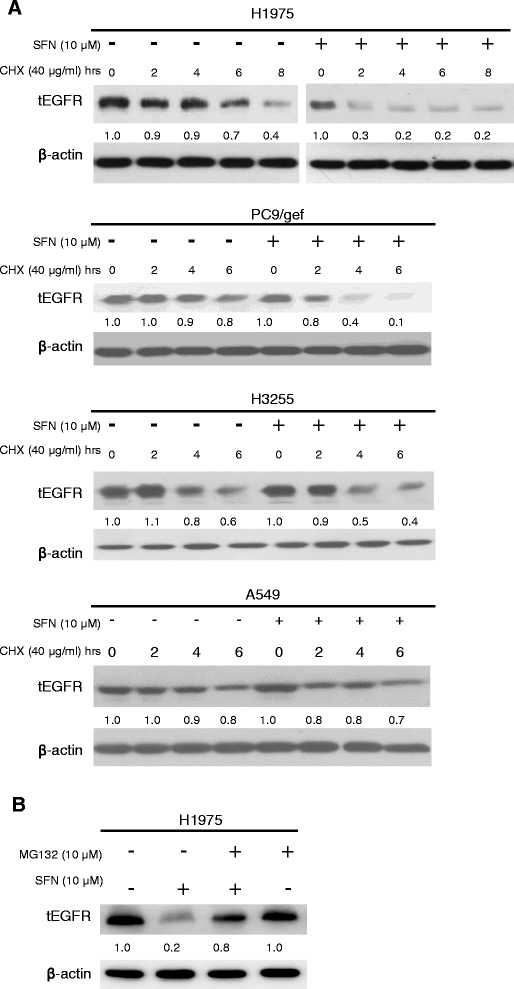
Effects of sulforaphane and MG132 on the stability of EGFR in NSCLC cells. (**a**) Cells were treated with or without 10 μM sulforaphane for the indicated durations in the presence of 40 μg/ml cycloheximide, and the level of tEGFR was determined by Western blotting. β-actin was used as the loading control. The relative levels of tEGFR at different times were quantified by densitometer and were shown below tEGFR. (**b**) H1975 cells were treated with 10 μM sulforaphane for 24 h in the presence or absence (control) of 10 μM MG132. The levels of tEGFR were assessed by Western blotting. β-actin was used as the loading control. The relative levels of tEGFR were quantified and shown below tEGFR. The data shown are representative of three experiments with similar results

As sulforaphane is a known proteasome activator [23], we next tested whether this agent could regulate EGFR-dependent signaling by affecting the stability of EGFR. Cells (H1975, PC9/gef, H3255 and A549) were treated with sulforaphane, protein synthesis was blocked by cycloheximide treatment, and the stability of EGFR was analyzed. As shown in Fig. 3a, the half-life of tEGFR in the absence of sulforaphane treatment was about 7.5 h in H1975 cells. In the presence of sulforaphane, the stability of EGFR was greatly reduced in H1975 and PC9/gef cells but only slightly reduced in H3255 and A549 cells. To address that the reduction of total EGFR (tEGFR) by sulforaphane was mediated through the proteasome, we examined the effects of a proteasome inhibitor (MG132) on the stability of EGFR in H1975 cells. As shown in Fig. 3b, the level of tEGFR in sulforaphane-treated cells was restored to about 80% when co-treated with MG132. Collectively, these results demonstrate that sulforaphane can stimulate the proteasome, thereby accelerating the degradation of EGFR in NSCLC cells.

### Sulforaphane enhances the antitumor activity of 17-AAG in NSCLC cells *in vitro* and *in vivo*

**Fig. 4:**
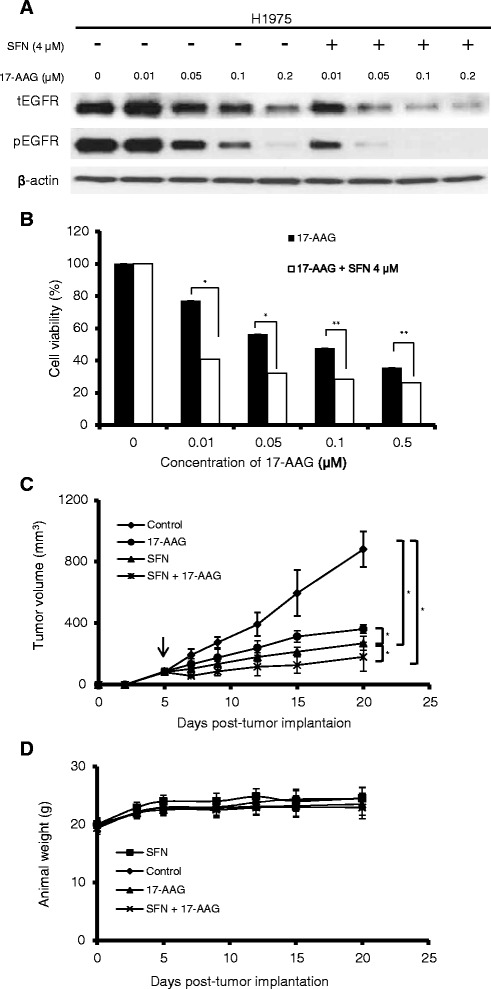
Effects of sulforaphane plus 17-AAG on tumor cell activity *in vitro* and *in vivo*. (**a** and **b**) H1975 cells were treated with the indicated concentrations of 17-AAG in the presence or absence of 4 μM sulforaphane for 24 h. The levels of total (t) and phosphoylated (p) EGFR were assessed by Western blotting (**a**), and the viability of the treated cells was assessed by the MTT assay (**b**). The data shown are representative of three experiments with similar results. (**c** and **d**) The effect of sulforaphane and 17-AAG treatment on subcutaneous xenografts of H1975 cells *in vivo*. H1975 cells were injected subcutaneously into the flanks of nude mice (*n* = 6 per group). Five days later (arrow), the mice were injected with PBS, 25 mg/kg 17-AAG, 10 μmol/kg sulforaphane, or a combination of sulforaphane and 17-AAG. Sulforphane was injected intratumorally five times per week. 17-AAG was administered intraperitoneally three times per week. Tumor volumes were determined twice weekly. The average tumors volumes (**c**) and body weights (**d**) were determined for each group. Results shown are the means ± SD of six mice; *p < 0*.*05; and **p < 0*.*01, as analyzed with the unpaired *t*-test

Heat shock protein 90 (HSP90) inhibitors reportedly have antitumor effects on TKI-resistant NSCLC cells harboring the T790M mutation in *EGFR* [24,25]. Our finding that sulforaphane enhances the degradation of EGFR (Fig. 2) prompted us to speculate that sulforaphane might prove useful as a single agent or as part of a combination therapy for the treatment of NSCLC harboring the EGFR T790M mutation. To test this hypothesis, we examined the efficacy of sulforaphane plus 17-AAG against H1975 cells *in vitro* and *in vivo*. To evaluate the antitumor effect of the combined treatment *in vitro*, we chose to use a suboptimal concentration of sulforaphane (4 μM) that had little or no effect on EGFR-related signaling (see Fig. 2). As shown in Fig. 4a, treatment of H1975 cells with 17-AAG alone dose-dependently reduced the levels of tEGFR and pEGFR. In the presence of 4 μM sulforaphane, treatment of H1975 cells with 17-AAG triggered much greater reductions in the levels of tEGFR and pEGFR. Similarly, the presence of 4 μM sulforaphane enhanced the cell-killing effect of 17-AAG, as assayed by the MTT method (Fig. 4b). These results indicate that combined treatment of 17-AAG plus sulforaphane appears to exert a synergistic effect (combination index < 1) on the cell viability and EGFR degradation of H1975 cells *in vitro*.

To investigate the antitumor effects of the combined treatment *in vivo*, we employed a xenograft animal model. Nude mice injected with H1975 cells developed tumors that reached ~ 100 mm^3^ in size after about 5 days. Beginning on day 5, the mice were treated with PBS, 25 mg/kg 17-AAG, 10 μmol/kg sulforaphane, or a combination of sulforaphane and 17-AAG. Sulforphane was injected intratumorally five times per week. 17-AAG was administered intraperitoneally three times per week. As shown in Fig. 4c, tumor growth was inhibited by the administration of 17-AAG or sulforaphane alone, and the combined treatment with sulforaphane plus 17-AAG produced an even greater inhibition of tumor growth. Under our experimental conditions, there was no noticeable change in body weight or overt sign of toxicity in the treated mice (Fig. 4d).

## Discussion

The receptor tyrosine kinase (RTK), EGFR, is a transmembrane protein with cytoplasmic kinase activity that transduces important growth factor signaling from the extracellular milieu to the cell. EGFR is frequently overexpressed and mutated in NSCLC [26]. Since many mutant EGFRs can activate signal transduction independent of ligand binding, *EGFR* mutations are strong predictors for the efficacy of EGFR-TK inhibitor (TKI)-based therapeutics. However, intrinsic and acquired resistance to EGFR-TKI remains a common phenomenon. To overcome the problems associated with EGFR-TKI resistance, strategies aimed at inhibiting EGFR signaling have been explored. As RTKs comprise the largest category of client proteins for HSP90 [7], one strategy aimed at targeting RTKs for degradation is to inhibit HSP90.

In view of the recent finding that sulforaphane can functionally regulate HSP90 [14-16], we postulated that this agent might attenuate EGFR signaling, and thus could prove useful for the treatment of TKI-resistant NSCLC. Here, we demonstrate that treatment with sulforaphane reduced viability and inhibited foci formation of TKI-resistant (H1975, PC9/gef, A549 and CL1-5) NSCLC cells (Fig. 1). H1975 cells, which harbor EGFR double mutations (L858R and T790M), were the most sensitive to sulforaphane treatment *in vitro* (Fig. 1). The sensitivity of TKI-resistant NSCLC cells to sulforaphane appears to be correlated with increased inhibition of EGFR-related signaling in these cells (Fig. 2). Although we do not yet know the detailed mechanisms underlying this increased inhibition of EGFR-related signaling, we found that sulforaphane appeared to decrease the stability of EGFR, possibly by increasing its proteasomal degradation (Fig. 3). In addition, we found that sulforaphane enhanced the degradation of total EGFR and phosphor-EGFR by 17-AAG (Fig. 4a). As 17-AAG is known to interact with the N-terminal nucleotide-binding domain of HSP90 (8) to exert its inhibition activity, it remains to be determined if sulforaphane may also interact with the N-terminal nucleotide-binding domain of HSP90. Previous studies have suggested that sulforaphane may inactivate histone deacetylase 6 (HDAC6)-mediated deacetylation of HSP90 [16], directly interact with specific amino acid residues of HSP90 and induce degradation of HSP90 client proteins [14], and/or activate the proteasomal system [23]. It is likely that the sulforaphane-induced modulation of EGFR stability observed herein may be attributed to one or more of these mechanisms.

Our finding of a novel role for sulforaphane in modulating EGFR led us to speculate that this agent might be capable of enhancing the therapeutic potential of other HSP inhibitors, such as 17-AAG, in treating TKI-resistant NSCLC. Indeed, we found that sulforaphane increased the antitumor activity of 17-AAG against TKI-resistant H1975 cells both *in vitro* and *in vivo* (Fig. 4). Therefore, sulforaphane may have potential as a nontoxic additive capable of increasing the therapeutic potential of other anticancer agents to treat NSCLC.

## Conclusions

In summary, we herein report that sulforaphane is a novel modulator of EGFR that destabilizes EGFR and down-regulates EGFR-related signaling in NSCLC cells. It is suggested that sulfornaphane should be further explored for its potential therapeutic application in the treatment of NSCLC.
